# Consumption of ultra-processed foods and risk for Alzheimer’s disease: a systematic review

**DOI:** 10.3389/fnut.2023.1288749

**Published:** 2024-01-15

**Authors:** Paola Alves Claudino, Nassib Bezerra Bueno, Sabrina Piloneto, Dieniffer Halaiko, Leticia Priscila Azevedo de Sousa, Cassia Helena Barroso Jara Maia, Bárbara Dal Molin Netto

**Affiliations:** ^1^Postgraduation Program in Food and Nutrition, Federal University of Paraná, Curitiba, Brazil; ^2^Postgraduation Program in Nutrition, Federal University of Alagoas, Maceió, Brazil; ^3^Pontifical Catholic University of Paraná, Curitiba, Brazil; ^4^Federal University of Paraná, Curitiba, Brazil; ^5^Postgraduation Program in Food and Nutrition, Department of Nutrition, Federal University of Paraná, Curitiba, Brazil

**Keywords:** ultraprocessed food, industrialized food, fast-food, Alzheimer’s disease, Alzheimer’s dementia

## Abstract

**Objective:**

To investigate the association of the consumption of ultra-processed foods with the risk of developing Alzheimer’s disease in adults and the elderly. The review protocol was registered on PROSPERO (CRD42022375944).

**Methods:**

This is a systematic review reported according to PRISMA guidelines. Observational studies were included without language or publication year restrictions. Studies assessing only other types of dementia as outcomes, not considering Alzheimer’s disease, were excluded. The research was carried out in the Medline, Embase, Lilacs databases, and a survey of the gray literature between April and November 2023, in addition to citation search in the included studies. Data extraction was performed by two independent reviewers. The risk of bias and methodological quality of the included studies were assessed using the Joanna Briggs Institute checklist for cohort studies.

**Results:**

A total of 5 studies involving 617,502 adults and elderly people were included. All studies had a cohort design and were considered of high methodological quality. Of the included studies, 4 demonstrated a risk association between the consumption of ultra-processed foods and the development of Alzheimer’s disease, while 1 study showed a risk association only with the development of cognitive decline.

**Discussion:**

The association between ultra-processed foods consumption and the risk of developing Alzheimer’s disease is a recent topic in scientific studies, given that the oldest study identified by our review dates back to 2017. Of the four included studies, three showed a significant association between ultra-processed foods consumption and the risk of developing Alzheimer’s disease.

## 1 Introduction

With the global increase in life expectancy, concerns about diseases more prevalent among the elderly, such as dementia, are growing. In 2016, dementia affected approximately 44 million people worldwide, with projections indicating this number will rise to 135 million cases by 2050 (1, 2).

Alzheimer’s disease (AD) is the most common cause of dementia (2), characterized as a progressive physiopathological restructuring of the brain, resulting from the extracellular deposition and accumulation of β-amyloid protein in the cerebral parenchyma (3). Currently, treatments to slow disease progression are limited, and the development of new drugs has been slow (4).

Evidence in the literature suggests that nutrition-related interventions are crucial for preventing cognitive decline, as diet influences direct and indirect mechanisms that can modify AD risk (5). It’s important to note that due to nutritional transition, dietary patterns have dramatically changed over recent decades. Studies indicate a higher availability and consumption of high-energy-density foods and low concentrations of vitamins and minerals (6).

A significant factor in this scenario is the substantial increase in the consumption of ultra-processed foods (UPF) in worldwide. UPFs are products composed of various ingredients, many of which are exclusively industrial in use, resulting from a sequence of physical and chemical processes applied to foods and their constituents. These foods typically have higher levels of total fat, saturated fat, added sugar, energy density, and sodium, along with lower fiber and vitamin density (6). UPFs account for more than half of the total dietary energy consumed in developed countries like the USA, UK, and Canada (7, 8), and about a fifth of total dietary energy in middle-income countries such as Brazil, Mexico, and Chile (9–11). Information sources for estimating UPF consumption are based on dietary data, which can be assessed using tools like 24-h recall, food frequency questionnaires, and food diaries (12).

The relationship between the consumption of UPFs and adverse effects on brain health has been an area of growing interest in scientific research. Recent studies suggest that diets high in UPFs may be associated with lower cognitive performance, affecting abilities such as memory, attention, and reasoning (13–15). Furthermore, the presence of potentially inflammatory ingredients in UPF can trigger inflammatory processes in the body, including the brain, contributing to oxidative stress and cellular damage (16). There is also an observed potential link between the consumption of these foods and mental health disorders such as depression and anxiety (16–19).

Recently published, a prospective cohort study revealed that UPFs consumption is associated with a higher risk of AD (20). There are various biological mechanisms that may explain this association. Among them, UPF is generally associated with low-quality diets, as they are rich in sugar, fat, sodium, and chemical additives (21). This combination of characteristics can promote systemic inflammation in the body and favor neurogenerative and pathophysiological processes in the brain, consequently increasing the risk of AD (22, 23).

Despite the well-established impact of UPFs consumption on brain health in the literature, the association with the risk of AD has been relatively underexplored by systematic reviews. Therefore, the objective of this present review was to investigate the association between UPFs consumption and the risk of developing AD in adults and the elderly.

## 2 Methodology

The present study is a systematic literature review reported according to the Preferred Reporting Items for Systematic Reviews and Meta-Analyses (PRISMA) (22, 23), with its protocol registered in the PROSPERO database (CRD42022375944).

### 2.1 Eligibility criteria

The eligibility criteria for the studies were determined based on the PECO acronym. The study population included adults and the elderly who, at the beginning of the study, did not have Alzheimer’s or any other type of dementia. The exposure was the consumption of UPF. The comparator was considered to be the presence of a healthy dietary pattern composed of unprocessed or minimally processed foods, lower UPF intake, or the consumption of another food group. The outcome assessed was the presence or risk of developing AD.

We defined UPF according to the NOVA classification system, which includes products such as soft drinks, dairy drinks, fruit nectars, powdered mixes for fruit-flavored drinks, packet snacks, candies and chocolates, cereal bars, ice creams, packaged bakery, margarines and other butter substitutes, cookies or biscuits, cake mixes, breakfast cereals, pies, pre-prepared pasta dishes and pizzas, chicken and fish nuggets, sausages, hamburgers and other reconstituted meat products, instant noodles, and powdered mixes for soups or desserts preparation (24).

The inclusion criteria were observational studies, such as cross-sectional and cohort studies, prospective or retrospective, as well as case–control studies. There were no restrictions on type, language, and year of publication. Studies that assessed only other types of dementia as outcomes, such as senile dementia, vascular dementia, frontotemporal dementia, Parkinsonian dementia, and others, without considering AD in their analyses, were excluded.

### 2.2 Information sources

A search was conducted in the electronic databases Medline, Embase, and Lilacs, as well as a search in the gray literature in April and November of 2023. The search was last updated in the databases on November 20, 2023. In addition to the electronic search, reviewers also performed a citation search in the reference list of each included study, in order to identify potentially relevant studies that were not captured in the initial search. The principal investigator of the included studies, which did not have the full-text version available in the databases, was contacted via email to request its provision.

### 2.3 Search strategy

The search terms and strategy were developed in consultation with a research librarian from the Federal University of Paraná. We used terms from the Medical Subject Heading (MeSH), Health Sciences Descriptors (HCD), and keywords from articles identified in a previous search. The descriptors were Alzheimer’s disease, Alzheimer’s dementia, industrialized food, processed food, ultra-processed food, and fast food, combined using the Boolean operators AND and OR. The search strategy used in each database is presented in Table 1.

**Table 1:** Search strategy used in the review.

Date base	Search strategy
Medline	((((((“alzheimer disease”[All Fields]) OR (“alzheimer dementia”[All Fields])) AND (“industrialized foods”[All Fields])) OR (“processed food”[All Fields])) OR (“ultraprocessed food”[All Fields])) OR (“fast foods”[All Fields]))
Embase	(‘alzheimer disease’/exp. OR ‘alzheimer disease’ OR (alzheimer AND (‘disease’/exp. OR disease)) OR ‘alzheimer dementia’:ti,ab,kw) AND ‘industrialized foods’:ti,ab,kw OR ‘processed food’:ti,ab,kw OR ‘ultraprocessed food’:ti,ab,kw OR ‘fast foods’:ti,ab,kw
Lilacs	(doença de alzheimer) OR (enfermedad de alzheimer) OR (alzheimer disease) OR (demência de alzheimer) OR (alzheimer dementia) OR (demencia de alzheimer) AND (alimentos industrializados) OR (industrialized foods) OR (alimento processado) OR (processed food) OR (alimento ultraprocessado) OR (ultraprocessed food) OR (comida rápida) OR (fast foods)
Google scholar	Alzheimer disease OR Alzheimer dementia AND industrialized foods OR processed food OR ultraprocessed food OR fast foods

### 2.4 Study selection

After querying the databases, the articles were exported to the Rayyan^®^ software, with duplicates being removed first. The selection of eligible studies was independently carried out by two reviewers (P.C and S.P). The initial selection was made by reading the titles and abstracts. Subsequently, a full-text reading of the pre-selected studies was conducted. Finally, titles and abstracts from citations within the included studies were read, followed by a full-text reading of the pre-selected citations. The inclusion or exclusion of studies required consensus between the two reviewers, with any discrepancies being resolved by a third reviewer (C.M).

### 2.5 Data collection process

As a strategy to control measurement bias, data extraction was performed independently by two reviewers (P.C and S.P). Extracted data included authorship, year, location, study design, population, method for assessing UPF, tool for assessing AD risk, adjusted covariates, results, conclusion, and methodological quality. Any discrepancies in data extraction were resolved by a third reviewer (C.M).

### 2.6 Assessment of risk of bias and methodological quality

For the assessment of the risk of bias and methodological quality of the included studies, the Joanna Briggs Institute tool for cohort studies was used (25). The tool consists of 11 questions that were answered with yes, no, unclear, or not applicable. The questions in the tool evaluate selection, observation, and confounding biases. The instrument was independently applied by the two reviewers (P.C and S.P), and discrepancies were resolved in consensus with a third reviewer (C.M). The studies were classified as follows: high methodological quality (≥5 “yes” answers), moderate methodological quality (3–4 “yes” answers), or low methodological quality (0–2 “yes” answers) (25). The potential biases identified in each study were evaluated descriptively. All selected studies, regardless of methodological quality, were submitted to data extraction and synthesis.

## 3 Results

### 3.1 Database search and study selection

**Figure 1:**
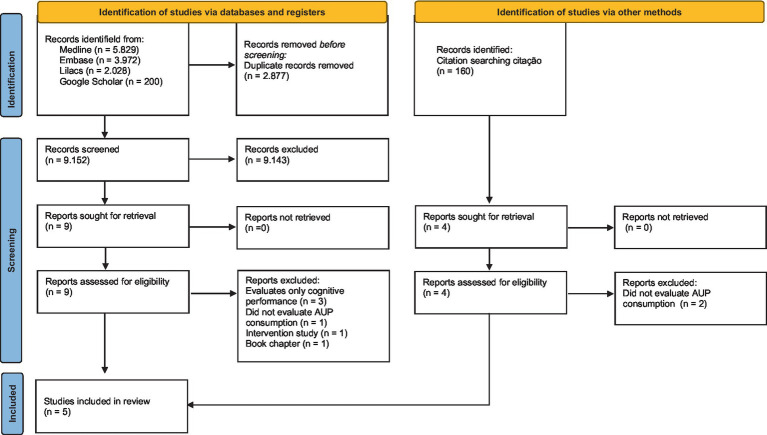
Flowchart of study selection.

The initial search yielded 12,029 records. After removing duplicates, 9,152 studies were screened based on title and abstract, with 9 studies retained for full-text evaluation. Considering the eligibility criteria, 3 studies were included in the review. From the included studies, 160 citations were identified in the references; of these, 4 were selected based on title and abstract, with only 2 being included. In total, 5 studies were included in the review. The steps of the study selection process are presented in the Flowchart (Figure 1).

### 3.2 Characteristics of the studies

The characteristics of the included studies are described in Table 2. All included studies had a cohort design. The studies were published between 2018 and 2023, they were conducted in the USA (26, 29), United Kingdom (13, 27), and Sweden (28). The duration of the monitoring period in the studies ranged from 8 to 24 years. In total, 617,502 individuals were monitored, with approximately 54.7% being female. The age of the participants ranged from 37 to 73 years.

**Table 2:** Characteristics of the included studies.

Author (Year; Location)	Study design/monitoring period	Population Age/sex (M/F)	UPF Method of consumption assessment	Tool Risk for AD	Covariates	Quality
Pase et al. (26) USA	Cohort/10 years Framingham Heart Study (2001–2011)	*N* = 1.484 Age: ≥ 60 (802/682)	Artificially sweetened soft drinks Sugary drinks Method: FFQ	Diagnostic and Statistical Manual of Mental Disorders, Criteria of the National Institute of Neurological, Communicative Disorders, and Stroke; Association of Disorders Related Criteria for AD, APOE genotyping	Age, sex, education level, total caloric intake, DGAI, SBP, HBP, CVD, atrial fibrillation, left ventricular hypertrophy, cholesterol levels, DM, alcohol intake, smoking, PA, positivity for APOE4 allele, waist-to-hip ratio	High
Zhang et al. (27) United Kingdom	Cohort/8 ± 1.1 years UK Biobank (2006–2015)	*N* = 493.888 Age: 40–69 (269.197/224.691)	Processed meats Method: FFQ/24HR	Hospital admission and death data, self-report, APOE genotyping	Region, BMI, PA, smoking, sleep duration, history of CVA, family history of dementia, consumption of vegetables and fruit, fish, tea, coffee, and alcohol	High
Samuelsson et al. (28) Sweden	Cohort/24 years Gothenburg (1992–2016)	*N* = 602 Age: 70 (384/218)	Western dietary pattern including UPF: processed meat, fast food, refined cereals Method: FFQ/portion sizes	Neuropsychiatric examination, neuropsychological test battery, APOE genotyping, information from relatives	Sex, total caloric intake, year of birth, education, PA, smoking, BMI, SAH, DM, serum cholesterol levels	High
Li et al. (20) United Kingdom	Cohort/12 years UK Biobank (2009–2021)	*N* = 118.528 Age: 37–73 (65.979/52.549)	Different UPFs: Sugar-sweetened beverages, processed dairy, snacks Method: 24HR NOVA classification	Hospital admission and death data	Age, sex, education, smoking, alcohol consumption, PA, BMI, sleep duration, CVD, family history of dementia, total caloric intake	High
Wang et al. (29) USA	Cohort/14.4 years Framingham Offspring	*N* = 2.909 Age: 54.2 ± 9.7 (1.555/1.354)	Different UPFs: fried food, processed meat, added sugar Method: FFQ Nova classification	Routine cognitive screening and comprehensive monitoring. National Institute of Neurological and Communicative Disorders and Stroke and the Alzheimer’s Disease and Related Disorders Association	SAH, DM, BMI, smoking, employment status, depression scale scores, personal income, marital status, education level, PA, serum cholesterol levels	High

BMI, Body Mass Index; CVD, Cardiovascular Disease; CVA, Cerebrovascular Accident; DM, Diabetes Mellitus; DGAI, Dietary Guidelines Adherence Index; FFQ, Food Frequency Questionnaire; PA, Physical Activity; SAH, Systemic Arterial Hypertension; SBP, Systolic Blood Pressure; 24HR, 24-h recall.

The studies considered various variables for analysis, such as age, gender, education, smoking, alcohol consumption, caloric intake, family history of dementia, history of stroke, diabetes mellitus, systemic arterial hypertension, cardiovascular disease, depression scale scores, atrial fibrillation, sleep duration, lipid parameters, physical activity, Body Mass Index (BMI), waist/hip ratio, and positive result for the APOE4 allele.

Regarding the tools for assessing AD risk, three studies highlighted the use of APOE genotyping (26–28). Other tools used were medical record reviews, neuropsychiatric test batteries, family self-reports, and standard manuals for AD diagnosis.

Considering the methods for evaluating UPF consumption, the study by Pase et al. (26) used a semi quantitative food frequency questionnaire (FFQ) that included 3 items about sugar-sweetened soft drinks, 4 items about fruit juices, 1 item about non-carbonated sugary beverages, and 3 items about artificially sweetened soft drinks. The FFQ was applied three times during the monitoring period. Zhang et al.’s study (27) used a FFQ covering 47 dietary items, including items related to processed and unprocessed meats, as well as the use of a 24-h recall (24hR), both applied at the beginning and end of the monitoring. Samuelsson et al.’s study (28) used the FFQ method and photographic records to estimate portion sizes at the start of the monitoring period. Li et al.’s study (20) used the 24HR method based on a web model, applied four times during the monitoring. Finally, Wang et al.’s study (29) using the validated 131-item Havard FFQ. The authors do not make it clear whether the QFA was applied more than once in the period (29). Only the study by Li et al. (20) and Wang et al. (29) used the NOVA system to classify the UPFs. The other included studies evaluated general UPF consumption (20, 29), Western diet consumption containing UPF (28), and specific UPF consumption, such as processed meats (27) and soft drinks (26).

### 3.3 Consumption of ultra-processed foods and risk for Alzheimer’s disease

The results of the included studies are presented in Table 3. The study by Li et al. (20), which evaluated the consumption of UPF using the NOVA classification, showed that higher intake of UPF was associated with an increased risk of incidence of AD. The replacing UPF with unprocessed or minimally processed foods was associated with a reduced risk of incidence of AD (20). Presenting similar outcome, the study by Wang et al. (29) showed that individuals who had higher consumption of AUP during the follow-up period were the group that exhibited the highest incidence of AD. Meanwhile, the study by Samuelson et al. (28), which compared a healthy dietary pattern with a Western-style diet, found that the group of individuals who consumed a Western diet containing UPF and who carried the APOE4 gene had a higher incidence of dementia; associations with the risk of AD were not found. The study by Zhang et al. (27), found that the group of individuals with higher consumption of processed meat showed a higher incidence of AD. Among these individuals, carriers of APOE4 had a significantly increased risk of developing AD, approximately 6 times higher compared to other groups. Lastly, the study by Pase et al. (26) which compared the consumption of artificially sweetened soft drinks with sugar-sweetened beverages, showed that the consumption of artificially sweetened soft drinks was associated with an increased incidence of AD Sugar-sweetened beverages were not associated with an increased incidence of AD (26).

**Table 3:** Results of the included studies.

Author	Results	Conclusion
Pase et al. (26)	Daily intake of artificially sweetened soft drinks was associated with an ↑ incidence of AD in models 1 and 2, adjusted for age, sex, caloric intake, education, IGAD, PA and smoking. However, these associations did not remain significant after adjustment for additional variables, involving SBP, SAH, CVD, atrial fibrillation, left ventricular hypertrophy, lipid profile, DM, waist-to-hip ratio and APOE4 positivity. Regarding recent beverage intake, daily consumption of artificially sweetened beverages ↑ the dementia incidence only in model 2. Neither fully sweetened beverages nor sweetened soft drinks were associated with dementia incident. DM status was identified as a partial mediator of the association between artificially sweetened beverage intake and AD	Consumption of artificially sweetened soft drinks was associated with a risk of development AD. Sugary drinks were not associated this outcome
Zhang et al. (27)	The group of individuals who had higher consumption of processed meat had a ↑ incidence of AD (HR: 1.52 per additional 25 g/day; 95% CI: 1.18, 1.96; P-trend = 0.001). Higher consumption of unprocessed red meat was associated with a ↓ incidence of AD of AD (HR: 0.70 per additional 50 g/day; 95% CI: 0.53, 0.92; P-tendency = 0.009). Among these individuals, APOE4 carriers had ↑ risks of developing AD by approximately 6 times. For APOE4 carriers, but not for non-carriers, there was a ↓ risk of AD with an increment of 50 g/day of unprocessed red meat	The consumption of processed meat might ↑ the risk of AD, with this risk being even ↑ in individuals with the APOE4 gene. The intake of unprocessed red meat might be associated with a ↓ risk of AD, regardless of the presence of the APOE4 gene
Samuelsson et al. (28)	There were interactions between dietary patterns and APOE4 status in relation to incident dementia (interaction value of p threshold <0.1), while no evidence of interactions was found between dietary patterns and NAD-PRSs. The group with higher adherence to a healthy dietary pattern showed a lower incidence of dementia among ε4 non-carriers (HR: 0.77; 95% CI: 0.61; 0.98), but not among APOE4 carriers (HR: 0.99; CI: 0.81; 1.21)	There is an interaction between the APOE4 status and adherence to dietary patterns in relation to incident dementia. Higher adherence to a Western dietary pattern was associated with an increased incidence of dementia among APOE4 carriers, but not among non-carriers. No evidence of interactions was found between dietary patterns and NAD-PRSs
Li et al. (20)	Adding 10% UPF to the diet was associated with a significant 13% increase in the risk of incidence AD. In addition, replacing 10% of UPF weight in diet with an equivalent proportion of unprocessed or minimally processed foods was estimated to be associated with an 17% lower risk of incidence dementia (HR: 0.83; 95% CI: 0.76, 0.91; *p* < 0.001). Among the UPF groups, the consumption of ultra-processed meat was associated with an ↑ risk of incidence AD (HR: 2.02; *p* < 0.001)	A higher intake of UPF was associated with a higher risk of incidence AD. Replacing UPF with unprocessed or minimally processed foods was associated with a decreased risk of incidence AD
Wang et al. (29)	During the follow-up of 14.4 years, a total of 306 incident dementia events occurred, including 184 (60.1%) cases of AD. The individuals in the highest quartile for energy-adjusted UPF consumption (over 9.1 servings per day) had a ↑ risk of AD dementia (HR: 1.75; 95% CI: 1.04–2.71) compared to the lowest quartile. A nonlinear dose–response pattern was shown for AD dementia	Higher consumption of UPF is associated with an increased risk of AD dementia

BMI, Body Mass Index; CVD, Cardiovascular Disease; DM, Diabetes Mellitus; DGAI, Dietary Guidelines Adherence Index; HR, Hazard Ratio; NAD-PRSs, Non-APOE AD polygenic risk scores; P, Percentile; PAF, Physical Activity; SAH, Systemic Arterial Hypertension; SBP, Systolic Blood Pressure.

### 3.4 Methodological quality and risk of bias

The assessment of the methodological quality of the included studies is presented in Table 4. Regarding the risk of bias, the study by Li et al. (20) did not clearly state in the methodology the confounding factors considered for outcome analysis, nor did it specify if there was sample loss during the monitoring period. The study by Zhang et al. (27) also did not clarify the confounding factors, and the studies by Pase et al. (26) and Samuelson et al. (28) did not specify about sample loss during the monitoring period. No bias was identified in the study by Wang et al. (29). Despite the presence of these mentioned biases (20, 26–28), all studies were considered of high methodological quality according to the JBI critical appraisal checklist for cohort studies.

**Table 4:** JBI critical appraisal checklist for cohort studies.

Question	Q1	Q2	Q3	Q4	Q5	Q6	Q7	Q8	Q9	Q10	Q11	Quality
Samuelsson et al. (28)	NA	NA	Y	Y	Y	Y	Y	Y	LC	LC	Y	High
Li et al. (20)	NA	NA	Y	LC	LC	Y	Y	Y	LC	LC	Y	High
Zhang et al. (27)	NA	NA	Y	LC	LC	Y	Y	Y	Y	NA	Y	High
Pase et al. (26)	NA	NA	Y	Y	Y	Y	Y	Y	LC	LC	Y	High
Wang et al. (29)	NA	NA	Y	Y	Y	Y	Y	Y	Y	NA	Y	High

^*^Yes (S); No (N); Lack of clarity (LC); Not applicable (NA).

## 4 Discussion

To the best of our knowledge, this is the first systematic review to investigate the association between UPF consumption and the risk of developing AD. Four studies provided evidence that UPF consumption was significantly associated with a higher risk of AD, while one study showed a risk association only with the development of dementia. However, these findings should be interpreted with caution due to the limited number of studies on the topic and the presence of heterogeneity among them. To classify foods according to the purpose and degree of processing, two study used the NOVA classification (17), introduced in Brazil in 2010, which categorizes foods and food products into four groups: unprocessed or minimally processed foods, processed culinary ingredients, processed foods, and UPF (30). Another study assessed the consumption of a Western diet characterized as being rich in UPF without using a standard dietary classification system (27). The other studies specified the type of UPF evaluated, being processed meats (27) and soft drinks and sugary beverages (26).

We observed that the use of different tools to assess dietary intake can be considered a bias for the analysis of the results. 24-h diet recalls, which were used in 2 studies of our review (17, 27), allow the inclusion of any and every reported food item and might better capture food intake by the degree of processing compared to food frequency questionnaires which contain predefined lists of food items (31). However, studies indicate that at least three repeated 24-h diet recalls are required to accurately represent habitual food intake (32). In our review, only the study by Li et al. (20) applied the 24-h diet recall considering this criterion, while Zhang et al. (27) applied the recall only on two occasions during the monitoring period. Considering that there are currently no validated dietary tools to capture UPF consumption, the development of tools specifically designed to assess the consumption of this food group is of great value to better investigate the association of UPF consumption with health outcomes (20).

Given the importance of considering the synergistic effects of foods, studying dietary patterns rather than isolated foods and nutrients is of great significance (33). The replacement of minimally processed foods and culinary preparations with ready-to-eat products is increasing worldwide, raising concerns for public health (23). It’s well-established in the literature that changes in population dietary patterns have been accompanied by an increase in the prevalence of obesity and other non-communicable chronic diseases (NCDs) (30). Previous studies have established a relationship between poor diet quality and increased mortality risk from NCDs (34). However, to date, no systematic review has specifically investigated the association of UPF consumption with the risk of developing AD.

Of the studies included in our review, Li et al. (20) in a cohort involving 118,528 adults and elderly individuals, found that higher consumption of UPF was associated with a higher risk of incidence of AD. The addition of 10% UPF in the diet was associated with a significant 13% increase in the risk of incidence of AD (20). Presenting similar outcome, the cohort study by Wang et al. (29) involving 2,209 adults, found that individuals who had higher consumption of AUP during the follow-up period were the group that exhibited the highest incidence of AD. Similar studies that evaluated UPF consumption, but only considering the outcome on cognitive function, showed that a higher percentage of daily energy consumption from UPF was linked to greater cognitive decline in adults and elderly individuals (33, 35). It’s important to note that, in our review, only studies assessing the risk of development AD were included since the pathophysiological basis of the disease differs from the pathophysiology of cognitive decline and other types of dementia, such as senile dementia, vascular dementia, or dementia in Parkinson’s disease. This distinction reduces potential biases in the outcome analysis.

In our sample, the study conducted by Pase et al. (26), which monitored a cohort of 1,484 elderly individuals, showed a significant association between the consumption of artificially sweetened beverages and a higher incidence of AD. On the other hand, no correlation was identified between the consumption of sugary drinks and incidence of AD. Other studies investigating the consumption of artificially sweetened beverages also demonstrated associations with health outcomes, such as a higher risk of cancer (35) and mortality from cardiovascular disease (36), however, results are controversial. More studies assessing the effect of consuming artificially sweetened beverages on health outcomes, including cognitive decline and AD, should be conducted.

The study conducted by Samuelson et al. (28) followed a cohort of 602 elderly individuals. Although it was the study with the smallest sample, it had a monitoring duration of 24 years, making it the longest cohort when compared to the other studies included in the review. Samuelson et al. (28) found that adherence to a Western dietary pattern containing UPF led to a higher incidence in the development of dementia only in individuals carrying the APOE4 allele.

Regarding the study by Zhang et al. (27) that tracked a cohort of 493,888 elderly individuals over 10 years, it was found that an additional consumption of 25 g/day of processed meats heightened the risk of incidence AD. Moreover, the AD risk was 6 times greater in individuals carrying the APOE4 allele (27). In the brain, APOE plays an important role in lipid transport to neurons, binding to APOE receptors on the cell surface involved in lipoprotein metabolism. Research has shown that modulation of these APOE receptors impacts amyloid and tau pathologies (37, 38). There are three predominant APOE alleles, the ε2, ε3, and ε4 alleles, which confer varying degrees of disease risk. APOE4 is a significant genetic risk factor for AD, while APOE2 nearly halves the AD risk and contributes to longevity (39).

It is also important highlighted that a significant volume of research has focused on impact of UPF consumption on cognitive function. A prospective study performed by Weinstein et al. (35), identified an association between high consumption of ultra-processed meats and accelerated cognitive decline in older individuals with type 2 diabetes mellitus. Another previous study demonstrated that an increase in UPF consumption was correlated with a higher risk of having short telomeres. This finding is pivotal, as short telomeres have often been linked to accelerated aging processes and subsequent cognitive impairment (40).

**Figure 2:**
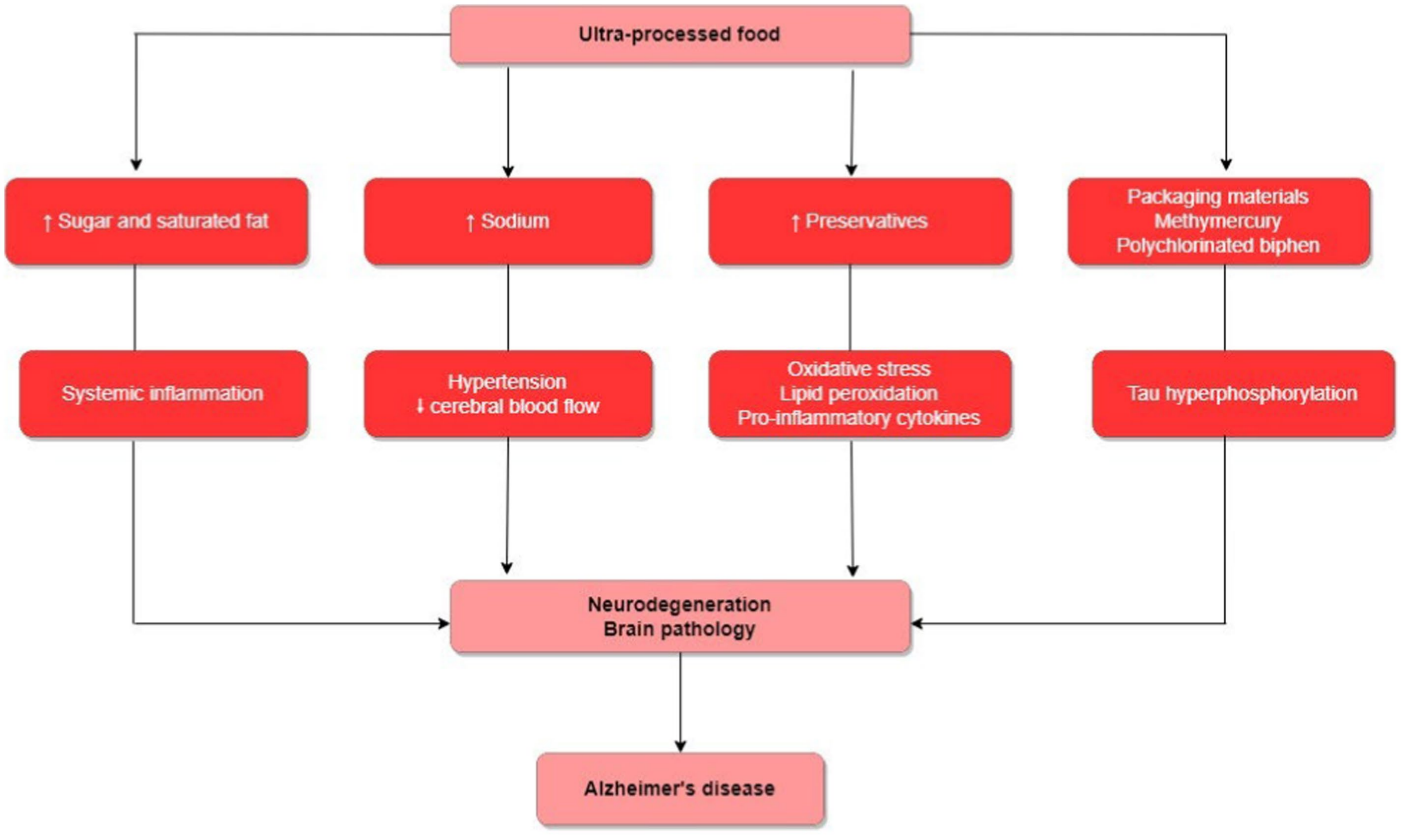
Biological mechanisms involved in the association of UPF consumption with the risk of developing AD.

In relation to the studies included in our review, all detail the potential mechanisms involved between the association of UPF consumption and the risk of AD (Figure 2). In general, UPFs are energy-dense, rich in refined carbohydrates, and saturated fatty acids (41). This combination of features can induce systemic inflammation, contributing to the progression of neurodegeneration and brain pathology (20, 26). Moreover, most UPFs, such as snacks, processed meats, and processed seasonings, are rich in sodium. High-sodium diets can cause systemic arterial hypertension, potentially accompanied by reduced cerebral blood flow, and possibly linked to the development of cognitive deficits (20, 27). Upon finding that processed meat consumption was associated with a higher risk of AD, Zhang et al. (27). argue that processed meats, like sausage, hot dogs, and salami, for instance, contain nitrites and N-nitroso compounds, which might lead to oxidative stress, lipid peroxidation, and activation of pro-inflammatory cytokines, among other mechanisms potentially involved in dementia development. Finally, UPF might be contaminated by packaging materials, some of which may have detrimental impacts on cognitive health, such methylmercury and polychlorinated biphenyl. An *in vivo* study suggested that methylmercury exposure induced tau hyperphosphorylation leading to neuropathological changes in the cerebral cortex selectively, which could increase the risk of AD (29). Regarding the mechanisms involved in the APOE-diet interaction in relation to the risk of AD, Samuelsson et al. (28) propose that the presence of the APOE4 allele is a risk factor for alterations in lipid and glycolytic metabolism, and when combined with a diet rich in ultra-processed foods, could heighten insulin resistance and inflammatory response, offering links to a greater risk of AD.

Also, it should be mentioned that there is some previous evidence regarding the consumption of UPF and the impact on the functionality of the Sirtuin 1 (SIRT1) gene. SIRT1 plays vital roles in cellular processes, such as stress response, energy metabolism regulation, inflammation modulation, and cellular aging (42). Studies indicate that the high content of fats and sugars in UPFs can compromise the functionality of SIRT1, disrupting metabolic and other cellular functions (43, 44). Another concerning factor is the presence of xenobiotics in UPF, which can interfere with the effects of SIRT1, contributing to disorders such as insulin resistance (43–45). It is worth noting that there is a growing convergence of evidence linking insulin resistance and metabolic dysfunction to the pathophysiology of AD (44–46).

Lastly, it is important to emphasize that the understanding that dementia has a prodrome spanning many decades, and that risk factors can exert effects well before the clinical diagnosis of the disease, is a burgeoning area of research in neurology (47). In line with this view, a standout prospective study by Sabia et al. (48) explored the impact of dietary patterns on the development of dementia over 25 years. The study demonstrated that risk factors for dementia influenced the disease’s trajectory many decades prior to diagnosis (48). Such findings underscore the importance of early preventive interventions and awareness of lifestyle modifications and diet has been frequently studied as an important part of this process.

Our review has several strengths. First, this review was the first to explore the association between UPF consumption and the risk of AD. We conducted an extensive literature search, including not only database searches but also gray literature and reference lists. Given that the included studies had a longitudinal design, it’s notable that our review showcased robust and impactful results. All the studies included were of high methodological quality.

Limitations should also be acknowledged. Of the four studies included in our review, two significant studies were found through citation search. This leads us to consider that the inclusion of more descriptors related to UPF might have expanded our search. It’s also important to note that no quantitative meta-analysis was conducted, an issue we hope will be surpassed in future research.

## 5 Conclusion

The association between UPF consumption and the risk of developing AD is a recent topic in scientific studies, given that the oldest study identified by our review dates from 2017. Out of the five studies included, four showed a significant association between UPF consumption and the risk of developing AD. Although there have been few studies on the topic so far, the included studies have a longitudinal design with robust results. Our findings reinforce the importance of public strategies aimed at raising awareness among the population about the harmful effects of UPF consumption on cognitive health.

## Data availability statement

The original contributions presented in the study are included in the article/supplementary material, further inquiries can be directed to the corresponding authors.

## Author contributions

PC: Writing – original draft. NB: Methodology, Supervision, Writing – review & editing. SP: Writing – original draft. DH: Writing – original draft. LA: Methodology, Writing – review & editing. CB: Writing – original draft. BN: Writing – review & editing.
